# Micro-nanoplastic induced cardiovascular disease and dysfunction: a scoping review

**DOI:** 10.1038/s41370-025-00766-2

**Published:** 2025-04-01

**Authors:** Adrian Goldsworthy, Liam A. O’Callaghan, Ciara Blum, Jarod Horobin, Lotti Tajouri, Matthew Olsen, Natalia Van Der Bruggen, Simon McKirdy, Rashed Alghafri, Oystein Tronstad, Jacky Suen, John F. Fraser

**Affiliations:** 1 Wesley Research Institute, Brisbane, QLD Australia; 2 Critical Care Research Group, The Prince Charles Hospital, Brisbane, QLD Australia; 3 Murdoch University, Perth, WA Australia; 4 Bond University, Gold Coast, QLD Australia; 5 Griffith University, Gold Coast, QLD Australia; 6 Perth Blood Institute, West Perth, WA Australia; 7 Dubai Police Scientific Council, Dubai, United Arab Emirates; 8 International Centre for Forensic Sciences, Dubai Police, Dubai, United Arab Emirates; 9 Physiotherapy Department, The Prince Charles Hospital, Brisbane, QLD Australia; 10 Institute for Molecular Bioscience, University of Queensland, Brisbane, QLD Australia

**Keywords:** Microplastic, Nanoplastic, Blood, Cardiovascular, Cardiac, Atherosclerosis

## Abstract

**Background:**

The human bioaccumulation of micro- and nano-plastics (MNPs) is increasingly being recognised in the aetiology and pathophysiology of human disease.

**Objective:**

This systematic scoping review aims to provide a comprehensive investigation of studies examining the impacts of MNPs on the human cardiovascular system.

**Methods:**

Five databases (PubMed, SCOPUS, CINAHL, Web of Science and EMBASE) were systematically searched.

**Results:**

Forty-six articles were identified, 13 of which investigated the presence of MNPs within the human cardiovascular system, including atherosclerotic plaques, saphenous vein tissue, thrombi and venous blood. The effect of MNPs on cell lines suggest MNPs are cytotoxic, immunotoxic, and genotoxic.

**Significance:**

The findings of this review, when evaluated together with additional studies utilising animal models, suggest MNPs may contribute to global cardiovascular morbidity and mortality. In particular, the ability of MNPs to induce endothelial damage, oxy-LDL formation, foam cell development and apoptosis, as well as to alter the clotting cascade, has potential implications for vascular diseases. In addition, MNPs may play a role in the aetiology and progression of congenital heart abnormalities, infective pathologies and cardiomyopathies. Despite an increasing awareness of the ability for MNPs to result in cardiovascular disease and dysfunction, a limited amount of research has been conducted to date characterising the presence of MNPs in the human cardiovascular system. Reseach is required to understand the extent of this rapidly emerging issue and to develop strategies that will support clinicians to appropriately manage and educate their patients in the future.

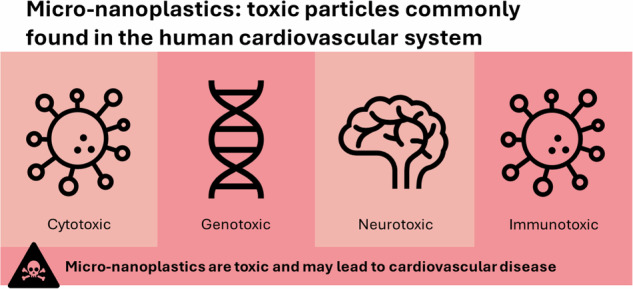

## Introduction

Cardiovascular disorders and environmental contamination from microplastics (MPs) are two major challenges within modern society [1, 2]. Currently, our understanding of the interrelationship between these two phenomena is limited [3]. Contemporary evidence points towards an increasing bioaccumulation of micro- and nano-plastics (MNPs) in humans, leading to increased disease and dysfunction within multiple organ systems, thereby presenting a threat to global public health [3, 4]. However, a lack of research and evidence synthesis to date currently leaves clinicians with insufficient data to guide the management of patients with MNP-associated disease and dysfunction.

In 2019, the World Health Organization (WHO) published a report entitled ‘Microplastics in Drinking-Water’, which minimised the significance of MNPs in drinking water in relation to their impact on human health [5]. The first report of MNPs in the human bloodstream was published in the same year. Since this time, the assertation that there is “no evidence to indicate a human health concern” [5], is increasingly being challenged by new studies. Once MNPs enter the human body, by means of inhalation, ingestion, or dermal absorption, they can cross biological barriers, leading to systemic exposure and bioaccumulation in vital organs and tissues [4, 6–8]. The ability of MNPs to influence inflammatory [6, 9], metabolic [6, 10], and endocrine pathways [11], in addition to their cytotoxic [12, 13], immunotoxic [6, 14], and genotoxic [12, 13] effects, suggests their implication in a number of disease processes. The discrepancy between the WHO report and current literature emphasises the need for urgent re-evaluation of the health impact of MNPs.

The definition of MNPs is a crucial starting point for this re-evaluation. A lack of consensus in literature elicits conflicts within public policy, legislation, research and medicine, compounding pre-existing challenges in monitoring and mitigating the impacts of MNPs. Moreover, the characteristics of these MNPs, such as their functionalisation, surface characteristics, shape, additives, pigmentation and polymer type, are essential in understanding their behaviour and impact on human health. However, these characteristics are yet to be considered in the literature, with most studies focusing only on particle size.

Cardiovascular disease remains a leading cause of morbidity and mortality globally, with data demonstrating that despite advances in recent decades, the mortality rate may be beginning to rise [1]. This concerning trend necessitates urgent research into the mechanisms surrounding the aetiology and progression of diseases relating to vascular pathologies, heart failure, and congenital and electrical abnormalities. This scoping review aims to systematically explore and summarise the literature surrounding MNPs in the human cardiovascular system and their pathological consequences, and explore the methodologies used in their detection and analysis, guided by the following research questions:

RQ 1. How are MNPs defined within the current cardiovascular literature?

RQ 2. What are the characteristics of plastics which have been found within human cardiovascular systems?

RQ 3. What methodology has been utilised to date to characterise plastics within human cardiovascular systems?

RQ 4. What are the pathophysiological considerations which have been explored regarding the presence of plastic in human cardiovascular systems?

For the purpose of this review, a broad definition of the term ‘cardiovascular system’ will be employed, inclusive of the heart, blood vessels, blood, and the components (e.g. immune cells) commonly found within human blood. While other reviews have previously provided broad insights into the potential health implications of MPs, this scoping review, through a rigorous and systematic interrogation of existing literature, attempts to solidify the field of knowledge surrounding MNPs and the cardiovascular system specifically, raise awareness of the scale of this emerging issue, and lay the foundation for further research which may assist in the development of health policies and clinical practice guidelines.

## Methods

### Protocol and registration

An a priori protocol was developed, informed by the recommendations of Arksey and O’Malley [15], the Joanna Briggs Institute (JBI) [16], and the PRISMA extension for scoping reviews reporting guidance (PRISMA-ScR). This protocol was published on the Open Science Framework (https://osf.io/w9hr5) on March 8th 2024. This review was conducted in accordance with the ethical principles set forth in the Declaration of Helsinki.

### Eligibility criteria

A pre-determined eligibility criteria was developed, informed by the population (human), concept (microplastics or nanoplastics and their effects) and context (cardiovascular system). Any studies investigating the presence of MNPs within the human cardiovascular system, or their effects on human cardiovascular outcomes or on relevant human cells lines, were included. Pre-determined definitions were developed and outlined within the a priori protocol after careful interrogation of the existing literature. For clarity, plastics were defined as a synthetic or semi-synthetic material comprising organic polymers from plant extracts or fossil fuels. The term ‘cardiovascular system’ was defined simply as the heart, blood, and associated vessels. To ensure a broad and thorough scope of the literature was undertaken, all research methodologies were included except for abstracts, reviews, pre-prints, conference proceedings, poster presentations, and editorials. No date restrictions were applied to the search strategy.

### Search strategy

A search strategy was developed utilising a three-step approach originally proposed by Arksey and O’Malley [15] and further outlined by the JBI. Firstly, a pilot search of PubMed and Google Scholar was undertaken on January 19th 2024. Secondly, results were reviewed to identify additional search terms, with the final search strategy being translated for additional search engines with the assistance of a validated search engine translation software (Systematic Review Accelerator [SRA] Polyglot) [17] (Appendix 1). The final search was executed on November 27th 2024. An additional search for grey literature was undertaken utilising Research Rabbit [18], TERA Farmer [19], and Perplexity [20].

### Information sources

Five databases (PubMed, EMBASE, CINAHL, SCOPUS, and Web of Science) were searched on November 27th 2024. Results from database searches were exported into Endnote X9 [21].

### Selection of sources of evidence

Duplicate results were removed utilising automation software (SRA Deduplicator) [22]. Articles were screened by two authors by title and abstract within SRA Screenatron [22]. Full text screening was undertaken within Covidence [23] by two authors with discrepancies resolved by a third author.

### Charting of data items

A draft extraction table was developed within Microsoft Excel to align with the aims of the scoping review. This was piloted and refined prior to undertaking full data extraction. Where information was not relevant or not reported, this was recorded for clarity.

### Synthesis of results

Data pertaining to definitions was extracted and, where possible, synthesised and visually represented. Similarly, data pertaining to the countries and years of publication was tabulated and visually represented. Studies identifying the presence of plastic in human specimens, and studies evaluating the effects of plastic on cellular viability, uptake, and function, have been tabulated separately.

## Results

### Selection of sources of evidence

**Fig. 1:**
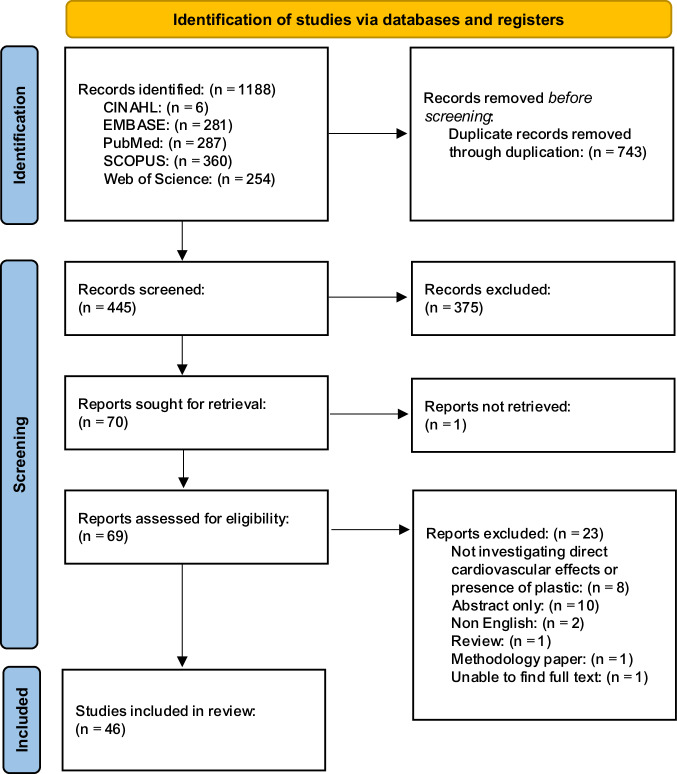
PRISMA ScR flow diagram.

Database searching led to the retrieval of 1188 articles, of which 743 articles were removed via automation within Systematic Review Accelerator and Covidence (Fig. 1). Title and abstract screening of the remaining 445 articles led to the exclusion of a further 375 articles. The full text of 69 out of the 70 identified articles was successfully retrieved and screened with substantial agreement between authors (Cohen’s Kappa = 0.760). This process led to the exclusion of a further 23 articles resulting in 46 articles being included within the review.

### Synthesis of results

**Fig. 2:**
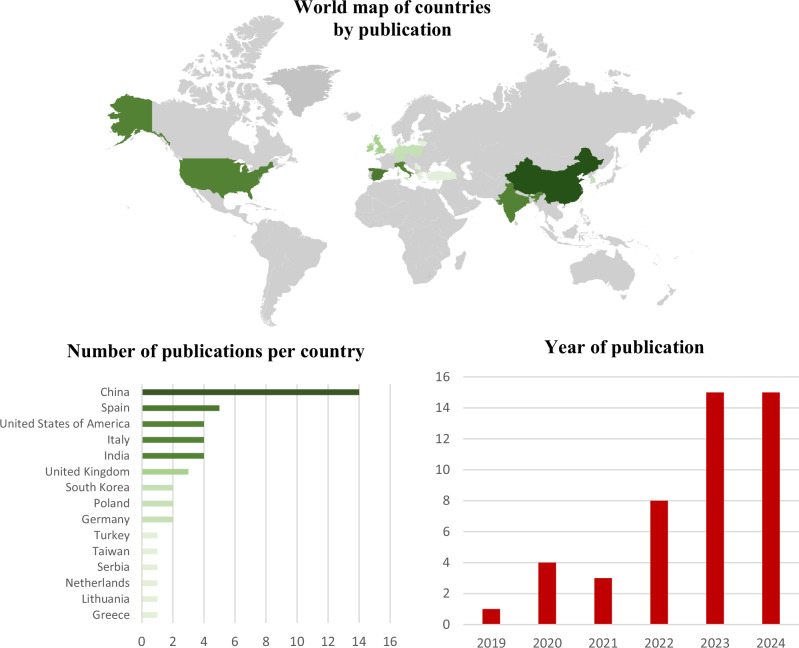
Details pertaining to country of origin, number of publications per country and year of publication.

**Fig. 3:**
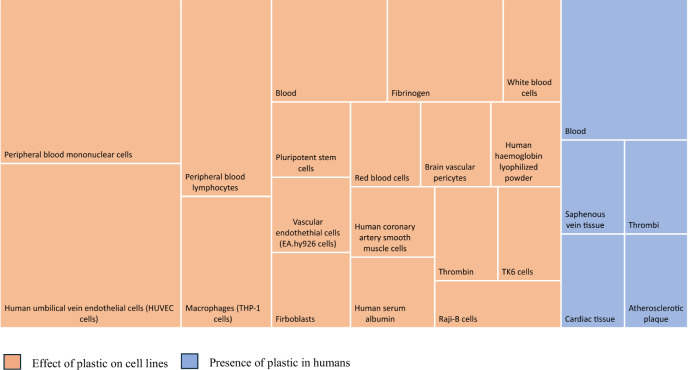
Treemap depicting proportion of studies reporting the presence and effect of plastics in human cells or tissues.

**Fig. 4:**
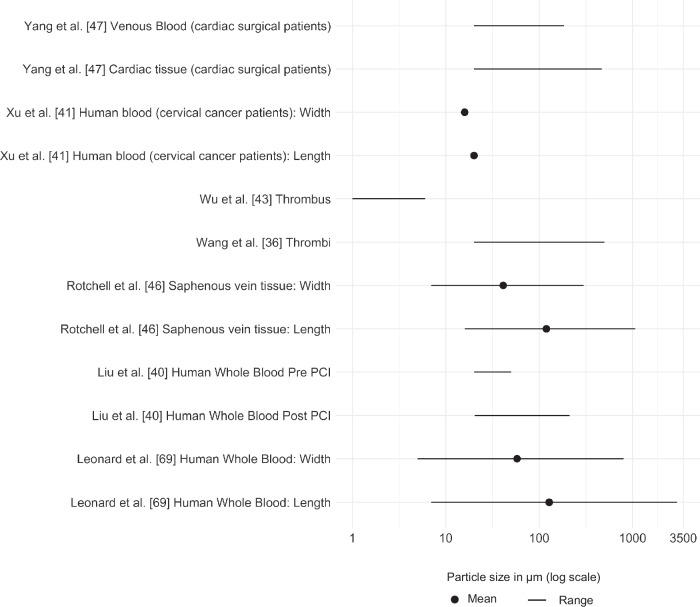
Size of plastic organised by sample type.

Of the 46 identified articles, 15 countries were represented, with China (*n* = 14), Spain (*n* = 5), the United States of America (*n* = 4), Italy (*n* = 4) and India (*n* = 4) representing over half (67%) of all identified publications (Fig. 2). Only one article was published prior to the WHO report on MPs in drinking water [5] being made publicly available. All articles defined MPs and nanoplastics (NPs) primarily based on the size of the particle (Table 1). Thirteen articles (Table 2) identified the presence of MNPs in venous blood samples, cardiac tissue, thrombi, saphenous veins and atherosclerotic plaques, with implications for all-cause mortality (Fig. 3). Sizes of identified particles varied greatly from 1 to 3000 μm (Fig. 4). The remaining 33 articles were in vitro investigations into the effect of MNPs on human cell lines relating to the cardiovascular system (Table 3). However, a discrepancy exists between the polymers used within in vitro studies and the types (Table 4) and characteristics of polymers that have actually been found in human vascular and cardiac tissue. The limited sensitivity of detection methodologies utilised to date has hindered the identification and characterisation of smaller NPs in human samples. These smaller NPs, as shown through in vitro studies, tend to have more pronounced adverse effects.

**Table 1:** Details pertaining to definitions of microplastics and nanoplastics listed by date of publication.

Author (Country)	Month, year of publication	Microplastic				Nanoplastic				Micro- and nano-plastics		
		<1 mm	<5 mm	100 nm –5 mm	1 µm – 5 mm	1 nm – 100 nm	< a few hundred nm	1 nm – 1000 nm	<100 nm	<1000 nm	< 5 mm	100 nm – 5 mm
Rubio et al. (Spain) [89]	April, 2020		✓									
Bojic et al. (United States of America) [24]	June, 2020	✓							✓			
Choi et al. (South Korea) [90]	June, 2020		✓									
Ballesteros et al. (Spain) [25]	August, 2020			✓					✓			
Cobanoglu et al. (Turkey) [13]	January, 2021			✓								
Lee et al. (United States of America) [48]	June, 2021		✓				✓					
Zhang et al. (China) [91]	November, 2021											
Leslie et al. (Netherlands) [26]	March, 2022		✓						✓	✓		
Fu et al. (China) [27]	April, 2022		✓						✓			
Lu et al. (China) [49]	July, 2022				✓					✓		
Wei et al. (China) [28]	August, 2022		✓						✓			
Malinowska et al. (Poland) [29]	November, 2022		✓						✓			
Tran et al. (United States of America) [30]	December, 2022	✓								✓		
Rotchell et al. (United Kingdom) [45]	February, 2023				✓							
Salvia et al. (Spain) [92]	February, 2023		✓					✓				
Wang et al. (China) [93]	March, 2023			✓		✓						
Wolff et al. (Germany) [77]	July, 2023		✓									
Yang et al. (China) [46]	July, 2023		✓									
Lu et al. (China) [47]	August, 2023										✓	
Gettings et al. (United Kingdom) [31]	October, 2023		✓							✓		
Ghosal et al. (India) [94]	November, 2023		✓									
Djapovic et al. (Serbia) [32]	December, 2023		✓							✓		
Wang et al. (China) [33]	January, 2024									✓		
Dailianis et al. (Greece) [53]	February, 2024		✓									
Leonard et al. (United Kingdom) [77]	May 2024				✓							
Liu et al. (China) [46]	May, 2024										✓	
Wang et al. (China) [35]	April, 2024										✓	
Xu et al. (China) [40]	June, 2024										✓	
Lomonaco et al. (Italy) [36]	June, 2024										✓	
Remigante et al (Italy) [37]	July, 2024										✓	
Yang et al. (China) [38]	August, 2024										✓	
Liu et al. (China) [39]	September, 2024										✓	
Lomonaco et al. (Italy) [36]	September, 2024		✓					✓				
Arranz et al. (Spain) [44]	October, 2024							✓				
Yu et al. (China) [95]	December, 2024								✓			✓
Hwangbo et al. (Republic of Korea) [96]	December, 2024							✓

**Table 2:** Details of articles investigating the presence of MNPs in samples from humans associated with the cardiovascular system.

Author (year) country	Sample type	Analysis approach	Characteristics of plastic		
			Quantity	Shape, size and colour	Polymer matrix
Leonard et al. United Kingdom [72]	Human whole blood	- Micro Fourier-transform infrared spectroscopy	- 2465.85 ± 4173.51 MP/L of blood	Shape: - Fragments Size: - Length: range 7–3000 μm, mean 127.99 ± 293.26 μm - Width: range 5–800 μm, mean 57.88 ± 88.89 μm Colour: White/clear (79%)	- Polyethylene - Ethylene propylene diene monomer - Ethylene-vinyl acetate/ethylene vinyl alcohol - Polyamide - Ethylene acrylic acid copolymer - Ethylene-butane copolymer - Polybenzimidazole - Polydimethylsiloxane - Polyethylene adipate diol - Polyethylene terephthalate - Poly(1-hexadecene) - Polyolefin - Polyoxymethylene - Polypropylene - Polyphthalamide - Polystyrene - Polyether urethane - Polyvinyl chloride - Resin - Vinylidene chloride-styrene Copolymer - Polyacrylamide - Polytetrafluoroethylene - Poly(3-hydroxybutyrate)
Leslie et al. Netherlands [26]	Venous blood	- Double shot pyrolysis gas chromatography/mass spectroscopy	- Average concentration 1.6 μg/mL	Shape: Not described Size: Not described Colour: Not described	- Polyethylene terephthalate - Polyethylene - Polystyrene - Polymethyl methacrylate - Polypropylene
Liu et al. China [34]	Human carotid and coronary arteries with atherosclerotic plaques, as well as the aorta without atherosclerotic plaques	- Pyrolysis gas chromatography/mass spectrometry	Coronary artery - Mean: 156.50 ± 42.14 μg/g tissue Carotid artery - Mean: 133.37 ± 60.52 μg/g tissue Aorta - Mean: 76.26 ± 14.86 μg/g tissue Overall findings - Mean concentration: 118.66 ± 53.87 μg/g tissue - Range: 52.62 to 225.23 μg/g tissue	Shape: Not described Size: Not described Colour: Not described	Coronary and carotid artery samples with atherosclerotic plaques: - Polyethylene terephthalate - Polyamide-66 - Polyvinyl chloride - Polyethylene Aorta samples: - Polyethylene terephthalate - Polyamide 66 - Polyvinyl chloride
Liu et al. China [39]	Human whole blood pre-percutaneous coronary interventions (PCI) and post-PCI	- Laser direct infrared spectrometry - Scanning electron microscopy	Pre-PCI - 4.96 ± 3.40 particles/10 mL of blood Post-PCI - 93.57 ± 35.95 particles/10 mL of blood Blood MP concentration over time: - 0 h post-PCI: 97.88 ± 33.74 particles/10 mL - 2 h post-PCI: 55.75 ± 13.09 particles/10 mL - 4 h post-PCI: 54.50 ± 20.80 particles/10 mL - 8 h post-PCI: 54.38 ± 18.64 particles/10 mL - 24 h post-PCI: 53.88 ± 19.13 particles/10 mL	Shape: - Rough - Irregular/fractured Size: Pre-PCI diameter - Range: 20.13–50.30 µm Post-PCI diameter - Range: 20.34–212.54 µm Colour: Not described	- Polyamide - Polyethylene - Polyurethane - Polyethylene terephthalate
Rotchell et al. United Kingdom [45]	Saphenous vein tissue	- Micro Fourier-transform infrared spectroscopy	- 14.99 ± 17.18 MNPs/g	Shape: - Irregular - Fibre Size: - Length: range 16–1074 μm, mean 119.19 ± 226.82 μm - Width: range 7–300 μm, 41.27 ± 62.8 μm Colour: Not described	- Alkyd resin - Polyvinyl propionate/acetate - Polyvinyl propionate ethylene - Nylon ethylene vinyl alcohol - Polyurethane
Salvia et al. Spain [92]	Venous blood	- Flow cytometry - Live cell analysis	Healthy: - Median: 614 events/μL - Range: 88–1460 events/μL Newborn: - Median: 562.1 events/μL - Range: 138.9–1038 events/μL Acute Lymphoblastic Leukaemia: - Median: 648.3 events/μL, - Range: 188–1354 events/μL Acute Myeloid Leukaemia: - Median: 577.2 events/μL - Range: 238.9–1274 events/μL Non-small cell lung cancer: - Median: 535.2 events/μL - Range: 101–992.6 events/μL Chronic lymphoid leukaemia: - Median: 536.5 events/μL, - Range: 123.3–1001.1 events/μL Idiopathic Nephrotic syndrome - Median: 556.4 events/μL, - Range: 353.6–1077 events/μL Multiple myeloma - Median: 500 events/μL, - Range: 138.0–1060.8 events/μL Type 1 diabetes mellitus - Median: 368.2 events/μL, - Range: 140.3–962.9 events/μL	Shape: Not described Size: Not described Colour: Not described	Not described
Wang et al. China [35]	Thrombi from thrombectomy procedures due to ischaemic stroke, myocardial infarction or deep vein thrombosis	- Pyrolysis gas chromatography mass spectrometry - Laser direct infrared spectrometry - Scanning electron microscopy	A total of 384 MNP particles detected in 24/30 (80%) of thrombi including: - Ischaemic stroke: 61.75 μg/g - Myocardial infarction: 141.80 μg/g - Deep vein thrombosis: 69.92 μg/g	Shape: Fragmented and spherical Size: - 20–50 μm (84.6%) - 50–100 μm (14.3%) - 100–500 μm (1.1%) Colour: Not described	- Polyethylene - Acrylate polymer - Polypropylene - Chlorinated polyethylene - Poly methacrylate - Polyurethane - Polyamide 66 - Polyvinyl chloride - Polyethylene terephthalate - Acrylate copolymer - Ethylene-vinyl acetate copolymer - Polybutadiene - Butadiene rubber - Ethylene-acrylic acid copolymer - Fluororubber - Methyl methacrylate-butadiene-styrene copolymer - Polyisobutylene - Polyoxymethylene
Wu et al. China [42]	Thrombus	- Raman spectroscopy	- Single particle	Shape: Not described Size: 1–6 μm Colour: Not described	- Polyethylene (low density)
Yang et al. China [46]	Cardiac tissue and venous blood samples from cardiac surgery patients	- Laser direct infrared - Scanning electron microscopy	Pericardium - Mean: 990,396.31 particles/g - Range: 1998.83–955,746.84 particles/g Epicardial adipose tissue - Mean: 79,980.47 particles/g - Range: 634.92–300,875 particles/g Pericardial adipose tissue - Mean: 19,201.42 particles/g, - Range: 19.69–77,843.58 particles/g Left atrial appendage - Mean: 22,574.73 particles/g, - Range: 164.11–65,625.00 particles/g Myocardium - Mean: 49.14 particles/g - Range: 9.79–88.50 particles/g - Venous blood (pre-surgery): - Mean: 7.2 particles/mL - Range: 2–14 particles/mL Venous blood (post-surgery): - Mean: 34.8 particles/mL - Range: 4–114 particles/mL	Shape: Particles, threads, rods Size: - Cardiac tissue: 20–469 μm - Venous blood: 20–184 μm Colour: Not described	- Polypropylene - Polyethylene terephthalate - Polystyrene - Polyethylene - Polyamide - Polycarbonate - Polyvinyl chloride - Polyurethane - Polymethyl methacrylate
Marfella et al. Italy [41]	Atherosclerotic plaque	- Pyrolysis gas chromatography/mass spectrometry - Stable isotope analysis	Atherosclerotic plaques: - Polyethylene: 21.7 ± 24.5 μg/mg - Polyvinyl chloride: 5.2 ± 2.4 μg/mg Out of 257 individuals: - 150 had polyethylene (58.4%) - 31 had polyvinyl chloride (12.1%)	Shape: Fragments with jagged edges observed Size: Not described Colour: Not described	- Polyethylene - Polyvinyl chloride
Xu et al. China [40]	Blood of human cervical cancer patients	- Raman spectroscopy - Pyrolysis-gas chromatography/mass Spectrometry	- 2.27 ± 1.48 MPs per mL	Shape: - Irregular - Fibre Size: - Average length 20.03 μm - Average width 15.93 μm Colour: Not described	- Polyethylene (23.68%) - Polyethylene-co-polypropylene (21.05%) - Polypropylene (13.16%) - Polyurethane (7.89%) - Polymethyl methacrylate (7.89%) - Rubber (5.26%) - Polyethylene vinyl acetate (2.63%) - Polyester (2.63%) - Polystyrene-co-polyvinyl chloride (2.63%) - Polyvinyl acetate (2.63%)
Yang et al. China [38]	Whole blood samples from patients presenting with chest pain.	- Pyrolysis gas chromatography/mass spectrometry	- Average concentration across all MP types was 150.08 µg/g of blood - Peaked at 413.99 µg/g of blood.	Shape: Not described Size: Not described Colour: Not described	- Polyethylene - Polyvinyl chloride - Polystyrene - Polypropylene - Polyamide 66
Yu et al. China [95]	Whole blood samples in patients with extracranial artery stenosis (ECAS) versus healthy controls	- Pyrolysis-Gas Chromatography/Mass Spectrometry - Laser Direct Infrared Spectroscopy - Scanning Electron Microscopy	- ECAS group: 174.89 ± 24.95 μg/g - Control group: 79.82 ± 31.73 μg/g	Shape: - Fragmented - Spherical Size: - 20–50 mm (77.9%) - 50–100 mm (17.1%) - 100–500 mm (5%) Colour: Not described	- Polyethylene - Polyurethane - Chlorinated polyethylene - Polyethylene terephthalate - Acrylate copolymer - Fluororubber - Polyvinyl chloride - Polymethyl methacrylate - Butadiene rubber - Polycaprolactone - Polyisobutylene - Polybutadiene - Polypropylene - Ethylene-vinyl acetate copolymer - Phenol-formaldehyde resin - Ethylene-acrylic acid copolymer - Polysulfone - Styrene-butadiene-styrene - Fluorosilicone rubber - Polylactic acid - Polymerised styrene butadiene rubber - Polystyrene - Acrylonitrile butadiene styrene - Styrene-isoprene-styrene triblock copolymer - Poly butylene adipate-co-terephthalate - Polycarbonate - Phenolic epoxy resin - Polyvinyl butyral - Methyl methacrylate-butadiene-styrene copolymer - Polyoxymethylene

*ECAS* extracranial artery stenosis, *HUVEC* human umbilical vein endothelial cells, *IL-6* interleukin-6, *MNPs* micro-nanoplastics, *MP* microplastics, *NF-κB* nuclear factor kappa-light-chain-enhancer of activated B cells, *NP* nanoplastics, *PBMCs* peripheral blood mononuclear cells, *PCI* percutaneous coronary interventions, *PMMA* polymethyl methacrylate, *PS* polystyrene, *RBC* red blood cells, *ROCK-1* rho-associated protein kinase 1, *ROS* reactive oxygen species, *RT-PCR* reverse transcription polymerase chain reaction, *TEM* transmission electron microscopy, *THP-1* human monocytic cell line derived from an acute monocytic leukaemia patient, *TNF-α* tumour necrosis factor-alpha, *VE-cadherin* vascular endothelial cadherin, *8-oxodG* 8-oxo-2’-deoxyguanosine.

**Table 3:** Details of articles investigating the effect of MNPs on human cell lines and blood samples associated with the cardiovascular system.

Author (year) country	Research aim/question/objective	Sample details	Analysis	Key findings
Arranz et al. Spain [44]	Aim: Characterise the effects of NPs in human whole blood from healthy donors.	Sample Type: Ex vivo human whole blood cells Plastic Model: - Size: 50–130 nm - Shape: Not described - Colour: Fluorescent and non-fluorescent - Polymer: Polystyrene, Polyethylene terephthalate, polylactic acid	- Scanning electron microscopy - Dynamic light scattering - Flow cytometry - Confocal microscopy - Dihydroethidium intracellular ROS assay - Plasma cytokine detection assay - Homolysis activity - Coagulation assay - Platelet activation assay	- NPs were internalised by all three assessed white blood cell types (monocytes, peripheral mononuclear cells and lymphocytes) within whole blood. - Internalisation rate dependent on cell type, particle type and particle characteristics such as charge. - Significant increases in pro-inflammatory cytokines following 24-h exposure including CXCL5. - High levels of haemolysis associated with polystyrene in comparison to other NP types. - No statistically significant difference in coagulation and platelet function.
Babonaitfó et al. Lithuania [51]	Aim: Evaluate the internalisation rates, cytotoxicity, and genotoxicity of polystyrene nanoparticles in human peripheral blood mononuclear cells in vitro.	Sample Type: - Peripheral blood mononuclear cells Plastic Model: - Size: 0.05–1 µm - Shape: Not described - Colour: Not described - Polymer: Polystyrene	- Flow cytometry - Density gradient centrifugation - AO/EB staining - Alkaline comet - Fluorescent microscopy - Cell viability - Micronucleus assay	- None of the tested nanoparticle concentrations had a cytotoxic effect on human peripheral blood mononuclear cells. - Increase in the levels of primary DNA damage after 24 h of exposure to polystyrene NPs in a dose-dependent manner. - All tested polystyrene NP concentrations induced a significant amount of micronucleated cells.
Ballesteros et al. Spain [25]	Aim: To assess genotoxic and immunomodulatory effects in human white blood cells after ex vivo exposure to polystyrene NPs.	Sample Type: - White blood cells Plastic Model: - Size: 0.04–0.1 µm - Shape: Not described - Colour: Not described - Polymer: Polystyrene	- Flow cytometry - Confocal microscopy - Comet assay - Indirect soft-agar assay - Cytokine quantification	- No significant cytotoxicity of polystyrene NPs in white blood cells. - Differential polystyrene NPs uptake among white blood cell lineages: ○ low in lymphocytes ○ high in monocytes ○ intermediate in polymorphonuclear cells - Increased DNA damage in monocytes and polymorphonuclear cells but not in lymphocytes. - Polystyrene NP exposure altered the blood secretome, increasing expression of cytokines related to inflammation, immune response, stress, and cell proliferation.
Bojic et al. United States of America [24]	Aim: Investigate the effects of polystyrene NPs on the transcription profile of preimplantation human embryos and human induced pluripotent stem cells.	Sample Type: - Pluripotent stem cells Plastic Model: - Size: 40, 200 nm - Shape: Spherical - Colour: Not described - Polymer: Polystyrene	- Pyrolysis-gas chromatography/mass spectrometry - Scanning electron microscopy - Confocal microscopy - Bulk RNA-seq - Principal component analysis - Gene set enrichment analysis	- Polystyrene particles were internalised by human embryos and human induced pluripotent stem cells. - Gene set enrichment analysis showed effects on atrioventricular valve development and the extracellular matrix in human induced pluripotent stem cells. - HiPathia analysis revealed altered signalling pathways and increased risk for ischaemic cardiovascular disease due to changes in lipid metabolism.
Chen et al. Taiwan [50]	Aim: Evaluate the toxicity of polystyrene MPs under realistic exposure levels in human vascular endothelial cells (EA.hy926).	Sample Type: - Vascular endothelial cells (EA.hy926) Plastic Model: - Size: 2.2–6.5 µm - Shape: Sphere - Colour: Not described - Polymer: Polystyrene	- Fluorescence microscopy - Trypan blue staining assay - JC-1 assay - ROS assay - Western blotting	- Polystyrene NPs induced oxidative stress by reducing antioxidant expression, leading to apoptotic cytotoxicity. - Heat shock proteins HSP70 and HSP90 were activated in response to oxidative stress induced by polystyrene NPs at cytotoxic levels. - Polystyrene NPs suppressed inflammation by inhibiting ROCK-1 and NF-κB p65 proteins at realistic blood concentrations. - Exposure to polystyrene NPs disrupted the vascular barrier by depleting the tight junction protein ZO-1, but did not significantly impact LOX-1 expression. - MNPs present a low risk of atherosclerosis.
Choi et al. Korea [90]	Aim: Investigate the chemical and physical toxicities of randomly-shaped polystyrene micro-fragments.	Sample Type: - Peripheral blood mononuclear cells - Fibroblasts Plastic Model: - Size: 5–25 µm, 25–75 µm, 75–200 µm - Shape: Random - Colour: Not described - Polymer: Polystyrene	- Cell viability - Cytokine release - Haemolysis and L-lactate dehydrogenase assay - ROS assay - Transmission electron microscopy	- Polystyrene micro-fragments can induce acute inflammation in immune cells, increase the production of ROS, and apoptosis in fibroblasts and cancer cells. - Physical stress caused by direct contact with micro-fragments can result in cellular membrane damage and haemolysis. - The physical damage to cells is amplified with increased concentration and roughness MNPs.
Christodoulides et al. United States of America [43]	Aim: Investigate the effect of MNP surface characteristics, concentrations and size on blood clotting dynamics.	Sample Type: - Venous whole blood Plastic Model: Size: 50–500 nm Shape: Spherical Colour: Not described Polymer: Polystyrene (non-functionalised, carboxylated, aminated)	- Thromboelastography	- Carboxylated polystyrene induced a clotting cascade, demonstrating increased fibrin polymerisation rates, and enhanced clot strength in a size and concentration dependent manner. - Non-functional polystyrene had minimal effects on clotting dynamics except for 50 nm particles at the lowest concentration. - The clotting effects of animated polystyrene (100 nm) resembled those of carboxylated polystyrene but were diminished in the 500 nm animated polystyrene group.
Cobanogul et al. Turkey [13]	Aim: Determine the genotoxic and cytotoxic effects of polyethylene MPs on peripheral blood lymphocytes.	Sample Type: - Peripheral blood lymphocytes Plastic Model: - Size: 10–45 µm - Shape: Spherical - Colour: Red - Polymer: Polyethylene (>70% polyethylene, <30% proprietary additive)	- Cytokinesis-block micronucleus assay	- Cytotoxicity in lymphocytes and increased level of genomic instability when treated with all MP concentrations for 48 h. - In vitro MP exposure significantly increased micronucleation, nucleoplasmic bridge formation and nuclear bud formation cytotoxicity in lymphocytes treated with five different MP concentrations for 48 h. - No decrease in the cell proliferation index, indicating a lack of MPs cytotoxic potential.
Dailianis et al. Greece [53]	Aim: Investigation of the cytogenotoxic and oxidative potential of both ultraviolet free and ultraviolet aged low density polyethylene MPs on healthy peripheral blood lymphocytes	Sample Type: - Peripheral blood lymphocytes Plastic Model: - Size: 100–180 µm - Shape: Not described - Colour: Not described - Polymer: Low density polyethylene	- Scanning electron microscopy - Fourier transform infrared spectroscopy - Wide angle X-ray diffraction patterns - Thermogravimetric analysis - Cell viability - ROS assay - Cytokinesis-block micronucleus assay	- Low density polyethylene MPs exposed to ultraviolet significantly decrease cell viability and increase ROS compared to pristine MPs. - Decomposition of the MPs’ surface enhances their bioreactivity towards lymphocytes. - Aged polyethylene MPs exhibited higher cytogenotoxic and oxidative effects compared to pristine ones.
Djapovic et al. Serbia [32]	Aim: Investigate the effect of polyethylene terephthalate nanoparticles on blood cells.	Sample Type: - Peripheral blood mononuclear cells Plastic Model: - Size: 300 nm - Shape: Sphere - Colour: Not described - Polymer: Polyethylene terephthalate	- MTT assay - Annexin V assay - ROS assay - Flow cytometry - Haemolysis	- Concentrations of nanoparticles <100 µg/mL have little effect on cell viability. - Haemolytic effects on RBCs - NPs might not be harmful, but the surfactants used in their production could have an impact.
Fleury et al. Germany [97]	Aim: Investigate the mechanical action of micron-sized MP particles with model cell membranes.	Sample Type: - Red blood cell Plastic Model: - Size: 0.8, 1, 8, 10 µm - Shape: Beads - Colour: Green (polystyrene), red (fluorescent polystyrene) - Polymer: Polystyrene, polyethylene and polymethylmethacrylate	- Fluorescence microscopy - Surface tension measurements - Theoretical modelling - Time to lysis following micropipette aspiration	- MP particles adsorbed on lipid membranes significantly increase membrane tension. - Each adsorbed MP particle consumes a surface area proportional to its contact area with the membrane. - Cumulative effect of MP particles leads to a reduction in membrane area and an increase in membrane tension. - MPs destabilise lipid bilayers via mechanical stretching, potentially leading to cell dysfunction. - MPs also destabilise red blood cells by mechanical stretching.
Fu et al. China [27]	Aim: Understand the cellular fate and toxicity of polystyrene and amino functionalized NPs to human umbilical vein endothelial cells.	Sample Type: - Umbilical vein endothelial cells Plastic Model: - Size: 50 nm - Shape: Not described - Colour: Not described - Polymer: Polystyrene and amino functionalised polystyrene	- MTT assay - Lactate dehydrogenase release assay - ATP activity assay - Quantitative real time PCR analysis - Live and dead cell staining - ROS assay - Mitochondrial membrane potential detection	- Positively charged polystyrene NPs are more toxic and display dose-related cytotoxicity. - Lactate dehydrogenase release was increased for functionalised polystyrene NPs compared to controls and control polystyrene NPs. - Functionalised polystyrene result in oxidative stress and induce damage to mitochondrial membrane potential, dysregulate mitochondrial dynamics, replication, and function-related gene expression. - ATP production decreased by 82% when exposed to 20 µg/mL (greater than non-functionalised polystyrene NPs).
Gettings et al. United Kingdom [31]	Aim: Evaluate the influence of MP and NP particles composed of polyethylene terephthalate on human brain vascular pericytes.	Sample Type: - Brain vascular pericytes Plastic Model: - Size: 82–250 µm - Shape: Not described - Colour: Not described - Polymer: Polyethylene terephthalate	- Seahorse XF cell mitochondrial stress test - Quantitative polymerase chain reaction - RT-PCR - ROS assay - Fourier-transform infrared spectroscopy - Thermogravimetric analysis - Differential scanning calorimetry	- The exposure of a monoculture of human brain vascular pericytes to polyethylene terephthalate particles in vitro at a concentration of 50 ppm for a duration of 6 days did not elicit oxidative stress. - Augmentation in various aspects of mitochondrial respiration, including extracellular acidification, proton pump leakage, maximal respiration, spare respiratory capacity, and ATP production in pericytes subjected to polyethylene terephthalate particles. - No statistically significant alterations in mitochondrial DNA copy number, or the expression of genes linked to oxidative stress and ferroptosis.
Ghosal et al. India [94]	Aim: Explore the interaction between polyethylene MPs and human haemoglobin.	Sample Type: - Human haemoglobin lyophilised powder Plastic Model: - Size: 34–50 µm - Shape: Not described - Colour: Not described - Polymer: Polyethylene (20–100 μg/mL)	- Ultraviolet-visible spectroscopy - Ultraviolet melting - Circular dichroism spectra - Fourier transform infrared spectroscopy - Thermal denaturing	- Polyethylene MPs bind to haemoglobin altering its associated proteins secondary structure through loosening and unfolding of protein architecture.
Gopinath et al. India [86]	Aim: Assess the physical changes in virgin-NPs during protein interaction, adverse effects of plasma coronated-NPs on human blood cells, toxicological impacts of virgin-NPs and the NPs isolated from cosmetics against human blood cells.	Sample Type: - Venous whole blood Plastic Model: - Size: 100 nm - Shape: Not described - Colour: Not described - Polymer: Polystyrene	- Serum albumin spectral analysis - Scanning electron microscopy - SDS-PAGE analysis - MTT assay - Comet assay - Haemolysis assay	- Several plasma proteins displayed strong affinity towards NPs and produced multi-layered corona of 13 nm to 600 nm. - The coronated-NPs often attracted each other via non-specific protein-protein attraction which subsequently induced protein-induced coalescence in NPs. - In the protein point of view, the interaction caused conformational changes and denaturation of protein resulting in bio-incompatibility. - The coronated-NPs with increased protein confirmation changes caused higher genotoxic and cytotoxic effect in human blood cells than the virgin-nanoparticles.
Hwangbo et al. Republic of Korea [96]	Aim: To evaluate the cytotoxic and inflammatory effects of fragmented polyethylene NPs on various human cell lines.	Sample Type: - Blood-derived cells (THP-1) Plastic Model: - Size: 25–350 nm, geometric mean diameter of 85.14 ± 5.37 nm. - Shape: Irregular fragments with uneven surfaces (non-spherical) - Colour: Fluorescent green - Polymer: Polyethylene	- MTS-based cell viability assay - LDH leakage assay - ELISA - Flow cytometry - Transmission electron microscopy	- Polyethylene NPs caused significant LDH leakage in THP-1 cells, indicating membrane disruption. - TNF-α levels were markedly elevated in THP-1 cells exposed to polyethylene NPs, suggesting an inflammatory response. - Polyethylene NPs accumulated in THP-1 cells, confirmed by increased fluorescence intensity and transmission electron microscopy imaging. - While polyethylene NPs did not significantly affect cell viability in the short term, they induced damage through membrane disruption and inflammation.
Koner et al. India [61]	Aim: Investigate the effect of polystyrene NPs on human macrophages.	Sample Type: - Macrophages (THP-1 cell line) Plastic Model: - Size: ≤450 nm - Shape: Not described - Colour: Not described - Polymer: Polystyrene (non-functionalised)	- Cell viability assay - Cellular proliferation assay - Fluorescent microscopy - ROS assay - Rh123 staining - Nuclear staining - Phase contrast microscopy	- Exposure of macrophages (THP-1) to polystyrene NPs: ○ Significantly decreased viability and proliferation ○ Increased oxidative stress ○ Resulted in morphological changes ○ Disrupted the mitochondrial membrane. ○ Induced apoptosis
Lee et al. United States of America [48]	Aim: Investigate the effect of polystyrene MPs on tube formation and cytotoxicity in human umbilical vein endothelial cells.	Sample Type: - Human umbilical vein endothelial cells Plastic Model: - Size: 0.5, 1, 5 µm - Shape: Not described - Colour: Not described - Polymer: Polystyrene	- Cell viability assay - Wound healing assay - Transwell migration ROS assay - Western blotting - Radioimmunoprecipitation assay - Bicinchoninic acid assay - SDS-PAGE analysis - Cell cycle analysis - Immunofluorescence	Treatment of human umbilical endothelial cells with polystyrene MPs: ○ Significantly decreased cell viability, with intracellular accumulation occurring in a dose- and size-dependent manner. ○ Increased autophagic and necrotic cell death. - Treatment of human umbilical vein endothelial cells with specifically 0.5 μm polystyrene MPs inhibited in a dose dependent manner: ○ Angiogenic tube formation ○ Angiogenic signalling pathways ○ Wound healing and cell migration
Li et al. China [54]	Aim: Investigate the effects of polystyrene NPs on cardiac differentiation and functionality of human embryonic stem cells.	Sample Type: - Human embryonic stem cells (hESCs) Plastic Model: - Size: 20 nm - Shape: Sphere - Colour: Not described - Polymer: Polystyrene	- Cell viability (CCK-8) assay - LDH assay - Flow cytometry - Western blotting - Transmission electron microscopy - Mitochondrial membrane potential assay (JC-1) - Mitochondrial density assay (Mito-Tracker Green/Red) - ROS assay - Alkaline phosphatase assay - RNA sequencing - qRT-PCR - Immunofluorescence - Calcium transient analysis	- Polystyrene NPs caused dose- and time-dependent cytotoxicity in hESCs, with decreased cell viability and increased LDH release. - Induced apoptosis with increased Bax and cleaved Caspase-3, decreased Bcl-2, and mitochondrial dysfunction. - Disrupted mitochondrial membrane potential, increased ROS. - Downregulated key pluripotency markers (SOX2, OCT4, NANOG), impairing stem cell self-renewal. - Reduced efficiency of differentiation into cardiomyocytes and cardiac organoids, with poorly organised sarcomeres and immature contractility, lower calcium transients, and reduced contractile function. - Activated p38/Erk MAPK pathways, contributing to pluripotency loss and impaired cardiac differentiation.
Lu et al. China [47]	Aim: Investigate the uptake and cytotoxic effects of polystyrene MNPs with a particle size of 1 μm on human umbilical vein endothelial cells in vitro.	Sample Type: - Human umbilical vein endothelial cells Plastic Model: - Size: 1 µm - Shape: Not described - Colour: Not described - Polymer: Polystyrene	- Cell viability assay - Lactate dehydrogenase release assay - Flow cytometry - Real-time quantitative PCR - Western blotting - Transmission electron microscopy - BCA protein assay kit - Confocal Microscopy - Immunofluorescent assay - ROS assay	- Interaction between human umbilical vein endothelial cells and 1 μm polystyrene MNPs was at a very low level even at high exposure concentrations of 25 μg/mL. - No significant differences in inflammation, autophagy, ROS level, lactate dehydrogenase release, and adhesion molecule expression following exposure to 1 μm polystyrene MNPs (5, 10, and 25 μg/mL) for 48 h. - Significant decrease in cell viability at the concentration of 100 μg/mL.
Lu et al. China [49]	Aim: Determine the interaction and autophagy effect of polystyrene NPs of sizes 100 nm and 500 nm on human umbilical vein endothelial cells.	Sample Type: - Human umbilical vein endothelial cells Plastic Model: - Size: 100, 500 nm - Shape: Spherical - Colour: Not described - Polymer: Polystyrene	- Cell viability assay - Flow cytometry - Scanning electron microscopy - Transmission electron microscopy - Lactate dehydrogenase release assay - Western blotting - Immunofluorescent assay - mCherry-GFP-LC3 lentivirus infection (assess autophagic flux level) - RNA extraction and real-time PCR - ROS assay	- Both 100 nm and 500 nm polystyrene NPs interacted with HUVEC in a time- and concentration-dependent manner. - 500 nm polystyrene NPs were bound to the surface of cell membranes, whereas 100 nm polystyrene NPs were internalised by HUVEC and aggregated in the cytoplasm. - Exposure to 25 µg/mL of 500 nm polystyrene NPs significantly increased lactate dehydrogenase release from HUVEC, indicating cell membrane damage. - Internalised 100 nm polystyrene NPs induced autophagy initiation and autophagosome formation in HUVEC. - Autophagic flux level was impaired in response to 100 nm polystyrene NPs, suggesting potential adverse effects on the cardiovascular system.
Malinowska et al. Poland [29]	Aim: Determine the genotoxic potential of non-functionalized polystyrene nanoparticles of different diameters (29, 44, and 72 nm) in human peripheral blood mononuclear cells in vitro.	Sample Type: - Peripheral blood mononuclear cells Plastic Model: - Size: 29, 44, 72 nm - Shape: Not described - Colour: Not described - Polymer: Polystyrene	- Cell viability assay - Comet assay - Fluorescence microscopy - Detection of 8-oxodG: 2D-UPLC-MS/MS analysis - Mass spectrometry - Field emission scanning electron microscopy	- Polystyrene NPs caused a decrease in human peripheral blood mononuclear cells metabolic activity, increased single/double-strand break formation, oxidised purines and pyrimidines and increased 8oxodG levels. - Resulting damage was completely repaired in the case of the largest polystyrene nanoparticles.
Martín-Pérez et al. Spain [98]	To determine how surface functionalization of polystyrene NPs affects their toxicokinetic and toxicodynamic interactions in human umbilical vein endothelial cells (HUVECs)	Sample Type: - Primary human umbilical vein endothelial cells (HUVECs) Plastic Model: - Size: 50 nm - Shape: Sphere - Colour: Fluorescent and non-florescent labelled - Polymer: Polystyrene (pristine, carboxylated, aminated)	- Transmission Electron Microscopy - Dynamic Light Scattering - Zeta Potential Measurements - Coulter Method - Flow Cytometry Confocal Microscopy - Dihydroethidium Assay - Comet Assay - Forward Scatter Analysis - Side Scatter Analysis	- All polystyrene-NPs were internalised by HUVECs, with carboxylated particles showing the highest uptake and aminated the lowest. - Aminated polystyrene -NPs caused the most significant effects despite slower internalisation. - Only aminated polystyrene -NPs significantly reduced cell viability. - All polystyrene -NPs induced ROS production, with aminated and carboxylated forms causing higher increases than pristine particles. - Carboxylated polystyrene -NPs significantly increased cell size and complexity, unlike aminated particles. - Pristine and carboxylated PS formed large vesicles, while aminated particles formed fewer and smaller vesicles. - Nanoparticles localised near the nucleus, suggesting potential interactions with genetic material. - Surface functionalization strongly influenced internalisation and biological effects.
Lomonaco et al. Italy [36]	Aim: Evaluate the effects of virgin and artificially aged polystyrene, high-density polyethylene, and low-density polyethylene MPs on the phenotype, metabolic activity, and pro-inflammatory status of human coronary artery smooth muscle cells.	Sample Type: - Human coronary artery smooth muscle cells Plastic Model: - Size: Polystyrene (average 564 µm), high-density polyethylene (average 622 µm), low-density (average 632 µm) - Shape: Not described - Colour: Not described - Polymer: Polystyrene, high-density polyethylene, low-density polyethylene (pristine and aged for 4 weeks at 40 °C with 750 W/m² simulated solar irradiation)	- Cell viability assay - ROS assay - Inflammasome and inflammatory markers analysis (Caspase-1, Interleukin-6 (IL-6) - Tumour necrosis factor-alpha (TNF-α) - Head-space sampling	- Virgin and artificially aged MPs induced oxidative stress and inflammatory responses. - Aged polymers showed significant overexpression of IL-6 and TNF-α, indicating activation of inflammatory processes. - Study identified type-specific volatile organic compound response profiles in vascular cells exposed to different MPs.
Płuciennik et al. Poland [99]	To evaluate the impact of non-functionalized polystyrene NPs with different diameters on human erythrocyte membrane fluidity, shape, and haemolysis.	Sample Type: - Human erythrocytes Plastic Model: - Size: 29, 44, 72 nm - Shape: Not described - Colour: Not described - Polymer: Polystyrene	- Haemolysis Assay - Electron Paramagnetic Resonance - Fluorescence Anisotropy Measurement (DPH and TMA-DPH probes) - Optical Microscopy - Zeta Potential Measurement - Dynamic Light Scattering - Atomic Force Microscopy - Scanning Electron Microscopy	- Smallest polystyrene NPs (29 nm) caused highest haemolysis, starting at 100 μg/mL. - Larger polystyrene NPs (72 nm) caused haemolysis at 200 μg/mL. - Stomatocyte formation increased with particle size, starting at 0.001 μg/mL (72 nm). - Membrane fluidity decreased, with significant changes at 0.001–0.1 μg/mL. - Negative zeta potential values influenced interactions; smaller particles (−29.68 mV) caused stronger effects. - Polystyrene NPs stiffened erythrocyte membranes, with smaller particles having greater impact. - Mechanical stress, not oxidative effects, caused shape changes. - Largest particles (72 nm) caused most shape alterations at low concentrations.
Remigante et al Italy [37]	Aim: Explore the effects of polystyrene MNPs on human erythrocytes, focusing on their internalisation, oxidative stress induction, and oestrogen receptor-mediated cellular responses.	Sample Type: - Human erythrocytes isolated from healthy non-smoker volunteers (male and female, aged 45–55) Plastic Model: - Size: MPs 1 µm mean diameter; NPs 0.10 µm mean diameter - Shape: Sphere - Colour: Fluorescent and non-fluorescent - Polymer: Polystyrene	- Haemolysis Assay - Scanning Electron Microscopy - Flow Cytometry - Confocal Microscopy - Western Blotting - ROS Detection - Thiobarbituric Acid Reactive Substances Assay - Sulfhydryl Group Content Measurement - Methemoglobin Assay - SO42− Uptake Assay - DIDS Inhibition Assay - Static Cytometry	- Polystyrene MNPs are internalised by human erythrocytes. - Internalisation is mediated by oestrogen receptors (ERα and ERβ) with evidence of ERα clustering on the plasma membrane. - Exposure to polystyrene MNPs induces oxidative stress, demonstrated by increased ROS production, lipid peroxidation, and protein sulfhydryl oxidation. - Erythrocyte morphology is altered, with increased acanthocytes, echinocytes, and leptocytes observed after exposure. - Polystyrene MNPs increase phosphorylation of ERK1/2 and AKT kinases, indicating activation of non-genomic pathways. - Band 3 protein levels decrease with polystyrene MNPs exposure, accompanied by clustering and altered SO42− ion exchange activity. - Polystyrene MNPs exposure leads to systemic concerns, including potential disruption of oxygen delivery and erythrocyte homoeostasis.
Rubio et al. Spain [89]	Aim: Determine the effects of polystyrene nanoparticles on different human leucocytic cell lines (Raji-B, TK6, and THP-1) in terms of cytotoxicity, cellular uptake, ROS production, and genotoxicity.	Sample Type: - Raji-B, TK6, THP-1 Plastic Model: - Size: 50 nm - Shape: Sphere - Colour: Not described - Polymer: Polystyrene	- Cell viability - Flow cytometry - ROS assay - Comet assay	- Polystyrene NPs were able to be internalised by all three cell lines, with THP-1 cells showing the highest particle internalisation. - No significant cytotoxicity effects were observed in any of the cell lines up to the concentration of 100 μg/mL. - Mild toxicity, ROS production, and genotoxicity were detected in Raji-B and TK6 cells, while no adverse effects observed in THP-1 cells.
Sarma et al. India [52]	Aim: Evaluate the genotoxic and cytotoxic effects of polystyrene NPs on human peripheral blood lymphocytes.	Sample Type: - Human peripheral blood lymphocytes Plastic Model: - Size: 50 nm - Shape: Not described - Colour: Not described - Polymer: Polystyrene (NP solution was sterilised by UV for 1 h before use)	- Cell viability assay - Haemolysis assay - chromosomal aberration assay - Cytokinesis-block micronucleus assay	- Polystyrene NPs showed dose-dependent cytolytic activity. - Reduction in cell viability. - Increased chromosomal aberrations and micronuclei formation was observed. - Findings indicate oxidative stress-mediated cytotoxicity, DNA damage and genomic instabilities.
Tran et al. United States of America [30]	Aim: Investigate the direct effects of MPs on fibrin clot formation using a simplified ex vivo human thrombin/fibrinogen clot model.	Sample Type: - Human plasma-derived fibrinogen and thrombin Plastic Model: - Size: 100 nm - Shape: Not described - Colour: Not described - Polymer: Polystyrene (functionalised & non-functionalised)	- Turbidity assay - Thromboelastography	- The presence of MPs decreases turbidity and the speed of fibrinogen conversion by thrombin. - Non-modified negatively charged particles have a greater effect than positively charged particles. - Non-modified negatively charged particles decreases the strength of the formed clots, primarily due to interactions with thrombin rather than with fibrinogen. - Protein coating of plastic particles modifies surface charge and limits further MP interactions with coagulation and fibrinolytic enzymes.
Wang et al. China [93]	Aim: Investigate the interaction of polystyrene NPs with human fibrinogen and its impact on protein structure and blood coagulation.	Sample Type: - Human fibrinogen Plastic Model: - Size: 80 nm - Shape: Sphere - Colour: Not described - Polymer: Polystyrene (non-functionalised & aminated)	- Transmission electron microscopy - Dynamic light scattering - SDS-PAGE assay - Ultraviolet–vis absorption spectroscopy - Fluorescence spectroscopy - Synchronous fluorescence spectroscopy - Aggregation experiments	- Polystyrene NPs disrupt the structure of human fibrinogen in a dose-dependent manner, driven mainly by hydrophobic forces. - NPs interacted with human fibrinogen similarly, with PS-NH2 having the greatest impact on human fibrinogen structure. - NPs have the potential to promote blood coagulation, with PS-NH2 again having a stronger effect.
Wang et al. China [33]	Aim: Investigate the effects of polystyrene NPs on the physiological functions of human serum albumin under physiological conditions and to study the interactions between polystyrene NPs and human serum albumin using multispectral methods and dynamic light scattering techniques.	Sample Type: - Human serum albumin Plastic Model: - Size: 80 nm - Shape: Sphere - Colour: Not described - Polymer: Polystyrene	- Esterase-like activity experiment - Equilibrium dialysis - Transmission electron microscopy - Fluorescence spectral measurements - Ultraviolet–vis absorption spectroscopy - Circular dichroism analysis - Dynamic light scattering	- Polystyrene NPs decrease the esterase activity altering the functional expression of human serum albumin - Higher amounts of polystyrene NPs increase the permeability of BPA, weakening human serum albumin-BPA interactions. - Stronger human serum albumin-polystyrene NPs binding at pH 4.0 increases the particle size of the polystyrene NPs-HSA complex. - The interaction between HSA and polystyrene NPs is characterised by static quenching, with electrostatic force as the dominant factor. - The study highlights the potential toxicity of NPs and the importance of understanding their interactions with blood proteins.
Wei et al. China [28]	Aim: Investigate the effects of anionic NPs, specifically polystyrene and poly(methyl methacrylate), on vascular endothelial cadherin junctions and endothelial leakiness.	Sample Type: - Human umbilical vein endothelial cells Plastic Model: - Size: 30, 50 nm - Shape: Sphere - Colour: Not described - Polymer: Polystyrene & polymethylacrylate	- Cell viability - Flow cytometry - ROS assay - Western blotting - RNA extraction and real-time quantitative PCR - Confocal fluorescence microscopy - Transwell assay	- Anionic polystyrene exposure induced endothelial leakiness mediated by conformational and structural changes to VE-cadherin junctions.
Wolff et al. Germany [77]	Aim: Measure the toxicity and activation of subtype differentiation to provide new insights into the potential risk of immune dysregulation caused by MPs exposure.	Cell Line: - Peripheral blood mononuclear cells Plastic Model: - Size: 20, 200 nm, 1 µm (unmodified polystyrene); 70, 400 nm, 1.1 µm (Polymethyl methacrylate) - Shape: Not described - Colour: Not described - Polymer: Surface unmodified polystyrene & polymethyl methacrylate	- Flow cytometry - Cytokine and chemokine multiplex analysis	- Pro inflammatory activation markers were decreased indicating a reduced innate immunity host defence capacity. - Aminated particles displayed greatest toxicity to immune cells and in particular macrophages

*ATP* adenosine triphosphate, *AKT* protein kinase B, *BPA* bisphenol A, *CXCL5* chemokine (C-X-C motif) ligand 5, *DNA* deoxyribonucleic acid, *DIDS* 4,4’-diisothiocyanatostilbene-2,2’-disulfonic acid, *EA.hy926* human vascular endothelial cell line, *ELISA* enzyme-linked immunosorbent assay, *ERK1/2* extracellular signal-regulated kinases 1 and 2, *HSA* human serum albumin, *HSP70* heat shock protein 70, *HSP90* heat shock protein 90, *HUVEC* human umbilical vein endothelial cell, *IL-6* interleukin-6, *LOX-1* lectin-like oxidised low-density lipoprotein receptor-1, *MAPK* mitogen-activated protein kinase, *MP* microplastics, *MNP* micro-nanoplastics, *NF-κB* nuclear factor kappa-light-chain-enhancer of activated B cells, *NP* nanoplastics, *PBMCs* peripheral blood mononuclear cells, *PMMA* polymethyl methacrylate, *PS* polystyrene, *RBC* red blood cells, *ROCK-1* rho-associated protein kinase 1, *ROS* reactive oxygen species, *RT-PCR* reverse transcription polymerase chain reaction, *SDS-PAGE* sodium dodecyl sulfate-polyacrylamide gel electrophoresis, *THP-1* human monocytic cell line derived from an acute monocytic leukaemia patient, *TNF-α* tumour necrosis factor-alpha, *VE-cadherin* vascular endothelial cadherin, *8-oxodG* 8-oxo-2’-deoxyguanosine.

**Table 4:** Visual display of the variety of plastics currently identified in human samples and utilised within cell line investigations.

		Utilised within cell line investigations																			Found in human samples				
		Peripheral blood mononuclear cells	Peripheral blood lymphocytes	Blood	Fibrinogen	White blood cells	Pluripotent stem cells	Red blood cells	Brain vascular pericytes	Human haemoglobin lyophilised powder	Vascular endothelial cells (EA.hy926 cells)	Human coronary artery smooth muscle cells	Thrombin	TK6 cells	Human umbilical vein endothelial cells	Human embryonic stem cells (hESCs)	Macrophages (THP-1)	Fibroblasts	Human serum albumin	Raji-B cells	Blood	Saphenous vein tissue	Thrombi	Cardiac tissue	Atherosclerotic plaque
Polymer matrix	Acrylate polymer																						**✓**		
	Acrylate copolymer																				**✓**		**✓**		
	Acrylonitrile butadiene styrene																				**✓**				
	Alkyd resin																								
	Butadiene rubber																				**✓**		**✓**		
	Carboxylated polystyrene			**✓**	**✓**										**✓**				**✓**						
	Chlorinated polyethylene																				**✓**		**✓**		
	Ethylene																								
	Ethylene acrylic acid copolymer																				**✓**		**✓**		
	Ethylene butane copolymer																				**✓**				
	Ethylene propylene diene monomer																				**✓**				
	Ethylene vinyl acetate/alcohol																				**✓**		**✓**		
	Ethylene-acrylic acid copolymer																				**✓**				
	Fluororubber																				**✓**		**✓**		
	Fluorosilicone rubber																				**✓**				
	Methyl methacrylate-butadiene-styrene copolymer																				**✓**		**✓**		
	Nylon ethylene vinyl alcohol																					**✓**			
	Phenol-formaldehyde resin																								
	Phenolic epoxy resin																				**✓**				
	Poly Butylene Adipate-co-Terephthalate																				**✓**				
	Poly(1-hexadecene)																				**✓**				
	Poly(3-hydroxybutyrate)																				**✓**				
	Polyacrylamide																				**✓**				
	Polyamide																				**✓**		**✓**	**✓**	**✓**
	Polybenzimidazole																				**✓**				
	Polybutadiene																				**✓**		**✓**		
	Polycaprolactone																				**✓**				
	Polycarbonate																				**✓**			**✓**	
	Polydimethylsiloxane																				**✓**				
	Polyester																				**✓**				
	Polyether urethane																				**✓**				
	Polyethylene		**✓**					**✓**		**✓**							**✓**				**✓**		**✓**	**✓**	**✓**
	Polyethylene co-polypropylene																				**✓**				
	Polyethylene (high density)											**✓**									**✓**				
	Polyethylene (low density)		**✓**									**✓**											**✓**	**✓**	
	Polyethylene adipate diol																				**✓**				
	Polyethylene terephthalate	**✓**		**✓**					**✓**												**✓**		**✓**	**✓**	**✓**
	Polyethylene vinyl acetate																				**✓**				
	Polyisobutylene																				**✓**		**✓**		
	Polylactic acid			**✓**																	**✓**				
	Polymerised styrene butadiene rubber																				**✓**				
	Polymethyl acrylates																								
	Polymethylmethacrylate	**✓**						**✓**													**✓**		**✓**	**✓**	
	Polyolefin																				**✓**				
	Polyoxymethylene																				**✓**		**✓**		
	Polyphthalamide																				**✓**				
	Polypropylene																				**✓**		**✓**	**✓**	
	Polystyrene	**✓**		**✓**	**✓**	**✓**	**✓**	**✓**			**✓**	**✓**	**✓**	**✓**	**✓**	**✓**	**✓**	**✓**	**✓**	**✓**	**✓**			**✓**	
	Polystyrene (aminated)			**✓**	**✓**										**✓**						**✓**				
	Polystyrene-co-polyvinyl chloride																				**✓**				
	Polysulfone																				**✓**				
	Polytetrafluoroethylene																				**✓**				
	Polyurethane																				**✓**	**✓**	**✓**	**✓**	
	Polyvinyl acetate																				**✓**				
	Polyvinyl butyral																				**✓**				
	Polyvinyl chloride																				**✓**		**✓**	**✓**	**✓**
	Polyvinyl propionate Acetate																					**✓**			
	Polyvinyl propionate ethylene																					**✓**			
	Resin																				**✓**				
	Rubber																				**✓**				
	Styrene-butadiene-styrene																				**✓**				
	Styrene-isoprene-styrene triblock copolymer																				**✓**				
	Vinylidene chloride-styrene copolymer																				**✓**				

### Definitions of microplastics and nanoplastics

Twenty-three (66%) articles provided a description of the term ‘microplastic’, 16 (41%) of the included articles defined the term ‘nanoplastic’, and nine utilised the descriptor MNP (25%). While earlier articles chose to define NPs as particles <100 nm in size [24–29], a shift occurred in 2022 whereby articles began to refer to NPs as particles less than 1000 nm in size [30–33] (Table 1). Increasingly, articles choose to refer to MNPs more generally as particles below 5 mm in size [34–40] while making specific reference to NPs as particles less than 1000 nm.

### Presence of MNPs in atherosclerotic plaques and thrombi and their effects on clotting factors

Two articles were identified analysing the presence of MNPs in atherosclerotic plaques [34, 41] and thrombi [42] respectively (Table 2). Of the 257 patients who completed the 33-month follow up, Marfella et al. [41] identified plastic (polyethylene) in carotid artery atherosclerotic plaques of 150 (58.4%) patients. Additionally, 31 (12.1%) patients had PVC in atherosclerotic plaques. At the 33-month follow up, patients with detectable MNPs had an increased risk of composite outcomes, including myocardial infarction, stroke, or death from any cause, compared to those with MNP-free atherosclerotic plaques [41]. Yang et al. [38] more recently explored the presence of MNPs within the bloodstream of patients with acute coronary syndrome. This study found MNPs within 100% of patients [38]. Similar to Marfella et al. [41], these results found that higher rates of MNP contamination were associated with poorer patient prognosis, as evidenced by higher SYNTAX scores, representing more complex and severe coronary artery atherosclerosis [38].

Two articles were identified which investigated the presence of MNPs in thrombi [35, 42]. Wu et al. published the first study identifying MNPs in human thrombi in 2023, in which they observed a single low density polyethylene particle, alongside other foreign materials including pigments, iron compounds, and metallic oxide particles [42]. Since this time, a larger study has provided further evidence of the widespread presence of MNPs in human thrombi, identifying 384 MNPs in 80% (24/30) of thrombi [35]. Several factors including particle size and functionalisation (e.g. carboxylated or aminated surfaces) have been shown to influence clotting dynamics, with smaller, functional particles demonstrating a greater ability to derange clotting dynamics under low shear environments [30, 43]. Conversely, Arranz et al. found no statistically significant difference in coagulation or platelet function with the addition of 50–130 nm sized polystyrene particles to ex vivo human whole blood at a concentration of 100 μg/mL [44]. While this study provided constant agitation, these in vitro conditions do not account for biochemical and biomechanical factors, such as shear stress, which influence clotting dynamics.

### Vascular tissue, endothelial cells and smooth muscle cells

A variety of polymer types have been reported within cardiac tissue obtained during open heart surgery and saphenous vein tissue, with significant variance in quantity per gram, shape and size [45, 46] (Table 2). When investigating the effect of MNPs on endothelial cells, identified articles commonly utilised human umbilical vein endothelial cells (HUVEC) [27, 28, 47–49] or vascular endothelial cells (EA.hy926) [50] (Table 3). Additionally, a single article by Lomonaco et al. [36] was identified, investigating the effects of polystyrene and polyethylene (both high and low density) on human coronary artery smooth muscle cells [36]. While Lu et al. [47] found little evidence of deleterious effects following exposure of HUVEC cells to 1 µm spheres, articles utilising smaller particle sizes found polystyrene MNPs to decrease cell viability and increase autophagy [48]. In particular, functionalised polystyrene particles were found to increase oxidative stress and lactate dehydrogenase (LDH), and induce mitochondrial damage, resulting in an 82% decrease in ATP production [27]. Similarly, aged MNPs significantly increased IL-6 and TNF, indicating increased inflammatory processes [36]. It is yet to be determined whether the increase in endothelial leakiness [28, 50] increases MNP interaction with vascular smooth muscle.

### Genotoxic effects

The exposure of polystyrene to peripheral blood mononuclear cells was shown to induce micronucleation and damage [25, 51]. While Ballesteros et al. [25] reported no DNA damage associated with 0.04 to 0.1 µm polystyrene NP exposure, Sarma et al. [52], utilising a particle size of 50 nm, demonstrated DNA damage and genomic instability. Dailianis et al. [53] demonstrated that exposure of low-density polyethylene to ultraviolet rays was associated with higher cytotoxicity and genotoxicity. Finally, Li et al. [54] identified 523 differentially expressed genes in response to polystyrene exposure. These genes are involved in processes such as cell development, mitochondrial and lysosomal function, and the downregulation of key pluripotency markers associated with reduced stem cell renewal efficiency.

## Discussion

### Overview

This systematic scoping review demonstrates that research into the presence and effect of MNPs in the human cardiovascular system has rapidly increased since 2019. While inconsistencies exist in the definition of MNPs in the early literature base, a consistent approach of defining MPs as particles less than 5 mm and NPs as less than 1000 nm in size has emerged since August 2023 (Table 1). The majority of included studies utilised in vitro experimental designs with human samples and cell lines. The findings of the 13 identified articles which investigated MNPs in human tissue are alarming and warrant concern from public health authorities. Of particular note is a lack of research into the presence of MNPs in human samples from low socioeconomic countries, especially those in the Pacific, which are economically and culturally tied to an ocean facing increasing contamination by MNPs. Taken together, the findings of research to date demonstrating the genotoxic, cytotoxic, immunotoxic and neurotoxic effects of MNPs, in addition to their deleterious effects on cellular metabolism and inflammatory effects, raise significant concerns for their role in a range of cardiovascular pathologies including atherosclerosis, cardiomyopathies, electrical and congenital abnormalities, and infective pathologies.

### The role of MNPs in atherosclerosis and coronary artery disease

In 2024, Marfella et al. [41] identified MNPs in 58.4% of atherosclerotic plaques, demonstrating that individuals with MNP-associated atherosclerosis had a higher rate of myocardial infarction, stroke, or death at 34-month follow up. Additionally, Yang et al. [38] identified a positive correlation between blood MP concentrations and coronary lesion complexity, as quantified by the SYNTAX (Synergy Between Percutaneous Coronary Intervention with Taxus and Cardiac Surgery) score. This study identified that acute coronary syndrome patients, particularly those with myocardial infarction, exhibited significantly higher microplastic burden, with associated elevations in inflammatory cytokines such as IL-6 and IL-12p70 [38]. Together, these studies highlight the concern that MNPs may not just play a role in the aetiology of atherosclerosis, but may actually be an important variable in understanding patient prognosis with implications for management decisions.

**Fig. 5:**
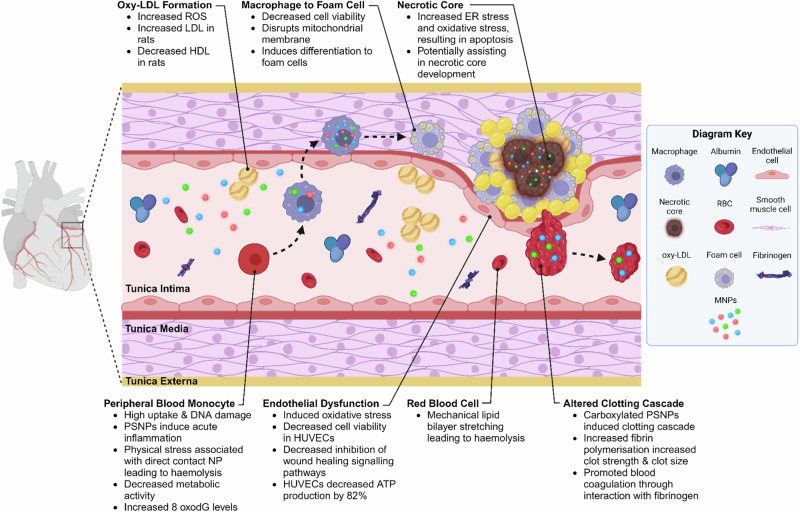
Pathways involving MNPs in the aetiology and pathophysiology of atherosclerosis. Created with BioRender.com.

Investigations employing human and animal cell lines have revealed a multitude of biochemical mechanisms, providing evidence for MNPs in the aetiology and pathophysiology of atherosclerosis, as well as for their significant role in vascular pathologies (Fig. 5). For example, MNPs have been demonstrated to induce endothelial dysfunction, an early stage of atherosclerotic plaque development [55]. Studies utilising 1 µm PS spheres have demonstrated little effect in human umbilical vein endothelial cell lines to date. In contrast, articles utilising smaller and positively charged particles, similar in size to those found within the observational study by Marfella et al. [41], have demonstrated increased ROS and LDH production. Additionally, studies have described damage to mitochondrial membranes, leading to a >82% decrease in mitochondrial ATP production [27], decreased cell viability and impaired angiogenesis, thereby hindering endothelial healing [48, 49]. In addition to endothelial dysfunction, MNPs have deleterious impacts within smooth muscle [56] and lead to decreased levels of high density lipoproteins (HDLs) as well as increased low density lipoproteins (LDLs) [57] and systemic ROS, assisting in the formation of oxy-LDL [58]. Taken together, these results demonstrate the ability of MNPs to lay the foundation for atherosclerotic plaque development.

Following their rapid uptake into the cytoplasm of macrophages, NPs provoke lipid aggregation [59], promoting the differentiation of macrophages into foam cells and the development of atherosclerosis [60]. Their continued genotoxic and cytotoxic effect from increased endoplasmic reticulum stress, oxidative stress and disruption to mitochondrial membranes [61] results in apoptosis [62], potentially assisting in the development of a necrotic core, increasing plaque instability [63].

In cases where plaque rupture ensues, MNP contamination deranges the clotting cascade, impacting fibrin polymerisation rates and platelet aggregation. This modulates clot strength and the manner in which the clot adheres to the endothelial wall [64]. Of particular clinical concern is the ability of MNPs to impede the production of endothelium-derived nitric oxide [58, 65], impairing vasodilatory responses to clot formation [66]. Importantly, SGLT2 inhibitors within porcine endothelial models treated with NPs have been shown to upregulate endothelial nitric oxide synthase expression, decrease the formation of ROS, and ultimately inhibit NP-associated endothelial cell senescence [67]. Together, these two studies demonstrate that the production of nitric oxide is perturbed by MNPs, which may impact the delicate haemostatic balance between thrombosis and bleeding. The many pathways through which MNPs may cause cardiovascular disease provide potential pharmacological targets, requiring further exploration into their pervasive effects. Regardless, the involvement of MNPs in atherosclerotic disease provides significant cause for concern, not only in the context of coronary artery disease, but also in peripheral and cerebrovascular pathologies [41].

### Valvular disorders, cardiomyopathies, and electrical abnormalities

**Fig. 6:**
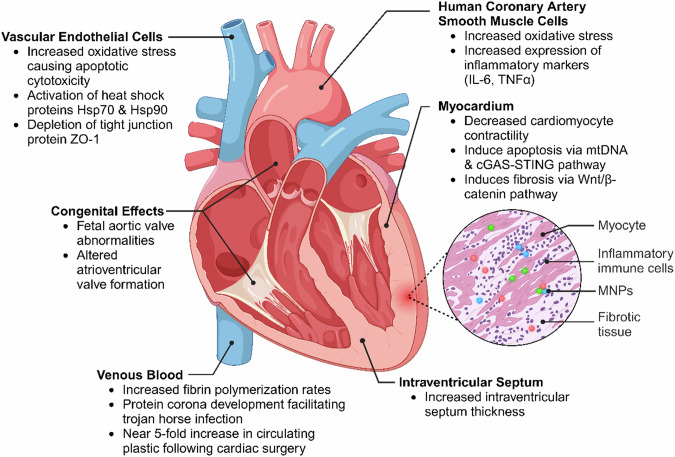
Effects of MNP on cardiac tissue. Created with BioRender.com.

In addition to vascular diseases, MNPs have been implicated in the dysfunction of cardiomyocytes [68] with potential implications for cardiomyopathies and electrical abnormalities [54, 56, 69] (Fig. 6). For example, the exposure of neonatal ventricular myocytes to NPs has been shown to significantly decrease intracellular Ca^2+^ levels, in addition to mitochondrial membrane potentials and cellular metabolism, resulting in a reduction in cardiomyocyte contraction forces [69]. Additionally, MNPs in rat models have been shown to induce cardiac fibrosis through activation of the Wnt/β-catenin pathway and cellular apoptosis [70]. Following polystyrene exposure, in vivo rat models have demonstrated increased troponin I and creatine kinase-MB (CK-MB) levels, as well as disruption of mitochondrial mtDNA and cGAS-STING signalling pathways, leading to cardiomyocyte apoptosis [68, 70, 71]. When exposed to MNPs at a concentration equivalent to human exposure, rats demonstrated a marked elevation in cardiac-specific markers and an increase in interventricular septal thickness [72]. This raises considerable concern and highlights a need for urgent research into MNP-associated cardiomyopathies [73].

### Cardiac disorders of infective origins

The rough surface characteristics and size of MNPs found within the human cardiovascular system to date [41] provide an ideal environment to facilitate the adsorption of viruses or bacteria, the development of biofilms, and increased virus survival and infectivity [74–76]. MNPs have been shown to promote the infection of cells through the development of a protein corona facilitating a trojan horse mechanism, whereby NP particles shuttle viruses and bacteria into the cytoplasm [77, 78]. Additionally, the presence of MNPs has been shown to inhibit innate immune functions, in particular the actions of macrophages [77, 78]. Beijer et al. [79] demonstrated a dose-related immune response with the largest secretions of IL-1β, IL-8 and TNF-α elicited by polyethylene terephthalate, identified within both human blood and cardiac tissue [26, 46]. As a result, MNPs are likely to play an important role in pathologies such as infective endocarditis, rheumatic heart disease and pericarditis.

### Congenital heart abnormalities

Of particular note, research highlighting the presence of MPs in human placentas (including on the fetal side), semen and the meconium of newborns raises important questions surrounding the potential role of MNPs in the aetiology of congenital cardiovascular abnormalities. Research investigating the potential abnormal development of the heart utilising pluripotent stem cells has demonstrated altered atrioventricular valve and cardiomyocyte formation following exposure to polystyrene NPs [80–82]. In animal models, NPs have been shown to alter umbilical and placental blood flow [83], with maternal polystyrene NP exposure leading to a 12% reduction in late gestational fetal weight [84]. With more specific reference to the cardiovascular system, maternal MP exposure in rats has also been observed to cause fetal aortic abnormalities [85]. Although current exposure levels are unlikely to cause significant cardiovascular anatomical or physiological abnormalities at birth, there is evidence that MNPs can affect cellular differentiation into cardiomyocytes, disrupt sarcomere organisation, impair contractility, and reduce calcium transients [54]. These findings raise concerns about potential subclinical alterations at birth that may contribute to clinical pathologies later in life [54].

### Current gaps in the literature

Despite significant advances in the field of MNPs and cardiovascular health, research is urgently required to assist in the characterisation of MNPs contaminating the human cardiovascular system. Currently, a lack of research exists to appropriately inform animal and cell line research regarding the characteristics of human environmental exposure (Table 4). Without a comprehensive understanding of the types, sizes, characteristics (leachates, surface characteristics, electrical charge, shape, etc.) and concentrations of MNPs within the human cardiovascular system, it is unclear if cell line research currently provides a solid understanding of the effects of MNPs within the general population or in specific populations, such as those investigated by Marfella et al. [41] (carotid endarterectomy) or Yang et al. [38] (acute coronary syndrome). Results of in vitro studies should, therefore, be interpreted with caution until further research characterises the presence of MNPs in humans and explores the long-term effects of their bioaccumulation on disease outcomes through additional in vivo studies which include long-term follow up. To assist with this, researchers moving forward should consider consulting with scientists familiar with the challenges associated with MNP detection and characterisation to ensure sensitive laboratory-based methodologies are utilised, thereby limiting the potential for false positives and environmental contamination.

In addition, researchers and public health authorities alike are urged to begin investigating the presence of MNPs in low socioeconomic areas, especially those identified as high risk due to exposure to contaminated water, food and living environments. Furthermore, the contamination of various clinical populations requires attention to understand variances in exposure and physiological consequences. In conjunction with laboratory-based analysis, complementary methodologies utilising surveys to characterise behaviour, alongside longitudinal studies within both animals and humans, are required to understand how various behaviours and exposures influence MNP contamination and its long-term effects on chronic disease and mortality. Clinical trials using behavioural interventions modifying MNP exposure, for example through dietary modifications, are urgently required to inform public health advice and international industry policy development. Additionally, research should seek to elucidate the potential impacts of specific environments (e.g. cities) and/or occupational hazards, especially in industries such as construction where individuals may be exposed to higher rates of MNPs associated with cardiovascular disease, such as poly vinyl chloride and polyethylene, as suggested by in vivo studies to date. An interdisciplinary approach which seeks to understand the multiple organ system interactions should be considered in order to advance our understanding of individual organ systems.

### Limitations

Due to the rapidly evolving nature of this research field, this scoping review will require updating within the next 2 years. At this time, further research that may allow for a systematic review and meta-analysis to be conducted on the presence of MNPs in various tissues is currently precluded by a lack of available data and consistency within methodologies and reporting. Additionally, a lack of research investigating the presence and effect of MNPs on the lymphatic system prohibits a robust discussion on how this complementary organ system affects cardiovascular function. Methodological limitations were noted within some articles which may have affected reported results. For example, reports of haemolytic activity may be overestimated considering Djapovic et al. [32] washed RBCs with hypertonic (0.99%) NaCl. Similarly, Gopinath et al. [86] isolated RBCs by centrifugation without a density gradient medium, which may have led to some leucocytes remaining with the RBC concentration, resulting in the release of haemolytic enzymes. Marfella et al. [41] highlighted the potential for laboratory contamination during MNP detection in atherosclerotic plaques, despite rigorous efforts to minimise this risk. They also noted that while pyrolysis-gas chromatography-mass spectrometry provides sensitive detection of MNPs, it does not differentiate between MPs and NPs, limiting precise characterisation of particle size and type.

## Conclusion

This systematic scoping review highlights the notable increase in research interest in this field since 2019, with all currently published studies reporting adverse effects on the cardiovascular system. Throughout their lives, humans are exposed to a multitude of MNPs with varying functionality, surface characteristics, chemical compositions and sizes every day. To date, research has identified the presence of MNPs within venous blood samples, cardiac tissue, thrombi, saphenous veins and atherosclerotic plaques, with implications for the prognosis of patients with cardiovascular disease and all-cause mortality. These findings, in conjunction with in vitro experimental designs, raise significant concern for the potential contribution of MNPs to cardiovascular pathologies such as atherosclerosis, cardiomyopathies, electrical abnormalities, congenital cardiovascular defects and infective pathologies. Multiple health authorities, including the WHO and the American College of Physicians, continue to call for urgent research in this field to elucidate the presence and effect of MNP bioaccumulation in humans, as well as to explore potential solutions [5, 87, 88]. Without further research, policy makers will be unable to act appropriately, and clinicians will lack the necessary guidance on how to assess, manage and educate their patients and the general public.

## Appendix 1

**PubMed**

(Microplastic*[tiab] OR Nanoplastic*[tiab])

AND

(cardiovascular[tiab] OR cardiac[tiab] OR blood[tiab] OR thrombi[tiab] OR artery[tiab] OR vein[tiab] OR microvascular[tiab] OR vascular[tiab] OR coronary[tiab] OR endothelial[tiab] OR clot*[tiab] OR thrombosis[tiab] OR pericard*l[tiab] OR endocard*[tiab] OR myocard*[tiab] OR adventitia[tiab] OR atherosclerosis[tiab] OR heart[tiab] OR cardiopulmonary[tiab] OR capillaries[tiab] OR bloodstream[tiab])

AND

(human*[tiab])

NOT

(Mice[tiab] OR Mouse[tiab] OR rodent[tiab] OR Rat[tiab] OR Rats[tiab] OR Murine[tiab] OR fish[tiab])

**EMBASE**

(Microplastic*:ti,ab OR Nanoplastic*:ti,ab)

AND

(cardiovascular:ti,ab OR cardiac:ti,ab OR blood:ti,ab OR thrombi:ti,ab OR artery:ti,ab OR vein:ti,ab OR microvascular:ti,ab OR vascular:ti,ab OR coronary:ti,ab OR endothelial:ti,ab OR clot*:ti,ab OR thrombosis:ti,ab OR pericard*l:ti,ab OR endocard*:ti,ab OR myocard*:ti,ab OR adventitia:ti,ab OR atherosclerosis:ti,ab OR heart:ti,ab OR cardiopulmonary:ti,ab OR capillaries:ti,ab OR bloodstream:ti,ab)

AND

(human*:ti,ab)

NOT

(Mice:ti,ab OR Mouse:ti,ab OR rodent:ti,ab OR Rat:ti,ab OR Rats:ti,ab OR Murine:ti,ab OR fish:ti,ab)

**SCOPUS**

(TITLE-ABS(Microplastic*) OR TITLE-ABS(Nanoplastic*))

AND

(TITLE-ABS(cardiovascular) OR TITLE-ABS(cardiac) OR TITLE-ABS(blood) OR TITLE-ABS(thrombi) OR TITLE-ABS(artery) OR TITLE-ABS(vein) OR TITLE-ABS(microvascular) OR TITLE-ABS(vascular) OR TITLE-ABS(coronary) OR TITLE-ABS(endothelial) OR TITLE-ABS(clot*) OR TITLE-ABS(thrombosis) OR TITLE-ABS(pericard*l) OR TITLE-ABS(endocard*) OR TITLE-ABS(myocard*) OR TITLE-ABS(adventitia) OR TITLE-ABS(atherosclerosis) OR TITLE-ABS(heart) OR TITLE-ABS(cardiopulmonary) OR TITLE-ABS(capillaries) OR TITLE-ABS(bloodstream))

AND

(TITLE-ABS(human*))

AND NOT

(TITLE-ABS(Mice) OR TITLE-ABS(Mouse) OR TITLE-ABS(rodent) OR TITLE-ABS(Rat) OR TITLE-ABS(Rats) OR TITLE-ABS(Murine) OR TITLE-ABS(fish))

**Web of Science**

((TI=Microplastic* OR AB=Microplastic*) OR (TI=Nanoplastic* OR AB=Nanoplastic*))

AND

((TI=cardiovascular OR AB=cardiovascular) OR (TI=cardiac OR AB=cardiac) OR (TI=blood OR AB=blood) OR (TI=thrombi OR AB=thrombi) OR (TI=artery OR AB=artery) OR (TI=vein OR AB=vein) OR (TI=microvascular OR AB=microvascular) OR (TI=vascular OR AB=vascular) OR (TI=coronary OR AB=coronary) OR (TI=endothelial OR AB=endothelial) OR (TI=clot* OR AB=clot*) OR (TI=thrombosis OR AB=thrombosis) OR (TI=pericard*l OR AB=pericard*l) OR (TI=endocard* OR AB=endocard*) OR (TI=myocard* OR AB=myocard*) OR (TI=adventitia OR AB=adventitia) OR (TI=atherosclerosis OR AB=atherosclerosis) OR (TI=heart OR AB=heart) OR (TI=cardiopulmonary OR AB=cardiopulmonary) OR (TI=capillaries OR AB=capillaries) OR (TI=bloodstream OR AB=bloodstream))

AND

((TI=human* OR AB=human*))

NOT

((TI=Mice OR AB=Mice) OR (TI=Mouse OR AB=Mouse) OR (TI=rodent OR AB=rodent) OR (TI=Rat OR AB=Rat) OR (TI=Rats OR AB=Rats) OR (TI=Murine OR AB=Murine) OR (TI=fish OR AB=fish))

**CINAHL**

(Microplastic*.tw. OR Nanoplastic*.tw.)

AND

(cardiovascular.tw. OR cardiac.tw. OR blood.tw. OR thrombi.tw. OR artery.tw. OR vein.tw. OR microvascular.tw. OR vascular.tw. OR coronary.tw. OR endothelial.tw. OR clot*.tw. OR thrombosis.tw. OR pericard*l.tw. OR endocard*.tw. OR myocard*.tw. OR adventitia.tw. OR atherosclerosis.tw. OR heart.tw. OR cardiopulmonary.tw. OR capillaries.tw. OR bloodstream.tw.)

AND

(human*.tw.)

NOT

(Mice.tw. OR Mouse.tw. OR rodent.tw. OR Rat.tw. OR Rats.tw. OR Murine.tw. OR fish.tw.)
